# Interactive effects of *OXTR* and *GAD1* on envy-associated behaviors and neural responses

**DOI:** 10.1371/journal.pone.0210493

**Published:** 2019-01-11

**Authors:** Toshiko Tanaka, Fumichika Nishimura, Chihiro Kakiuchi, Kiyoto Kasai, Minoru Kimura, Masahiko Haruno

**Affiliations:** 1 Center for Information and Neural Networks, NICT, Suita, Osaka, Japan; 2 Department of Neuropsychiatry, The University of Tokyo, Bunkyo-ku, Tokyo, Japan; 3 Brain Science Institute, Tamagawa University, Machida, Tokyo, Japan; Kanazawa University, JAPAN

## Abstract

Inequity aversion (negative feelings induced by outcome differences between the self and other) plays a key role in human social behaviors. The neurotransmitters oxytocin and GABA have been implicated in neural responses to inequity. However, it remains poorly understood not only how individual genetic factors related to oxytocin and GABA affect the neural mechanisms behind inequity aversion, but also how these genes interact. To address these issues, we examined relationships between genotypes, behavioral decisions and brain activities during the ultimatum game. We identified interactive effects between the polymorphisms of the oxytocin receptor gene (*OXTR*) and glutamate decarboxylase 1 gene for GABA synthesis (*GAD1)* on envy aversion (i.e., disadvantageous inequity aversion) and on envy-induced activity in the dorsal ACC (dACC). Thus, our integrated approach suggested interactive genetic effects between *OXTR* and *GAD1* on envy aversion and the underlying neural substrates.

## Introduction

Inequity aversion plays a key role in human social behaviors, such as cooperation and donation. Using economic games, functional magnetic resonance imaging (fMRI) studies have established that many brain regions, including the anterior cingulate cortex (ACC) [1–4], medial prefrontal cortex (mPFC) [1,5,6], dorsolateral prefrontal cortex (dlPFC) [1,3,7–9], insula [1,9], amygdala [2,10–11] and striatum [6–7,11], are involved in inequity aversion.

Over the last decade, the neuromodulator oxytocin has gained attention as an influencer on human social behaviors [12–24] and human emotional brain networks [25]. Oxytocin is a peptide hormone and neuropeptide produced in the hypothalamus. The axons of hypothalamic oxytocin neurons project to several regions associated with inequity aversion, including the amygdala, hippocampus, ACC and mPFC [26]. Several reports have investigated the effect of oxytocin on prosocial behaviors related to inequity aversion [27]. The administration of oxytocin was found to alter the subjective evaluation of unfairness [28] and money allocation with others in economic games such as the trust game and ultimatum game [13,17,24,29]. It is also reported that intranasal oxytocin increases envy (i.e., disadvantageous inequity) aversion and guilt (i.e., advantageous inequity) aversion [30]. In addition, relationships between the polymorphisms of the oxytocin receptor gene (*OXTR*) and social behaviors have been reported, including correlations of *OXTR* polymorphisms with trust and altruism [31] and with mental disorders such as autism [32–35]. However, the effects of *OXTR* polymorphisms on behaviors and brain activities associated with inequity aversion remain poorly understood [13,36].

GABA is the primary inhibitory neurotransmitter in the central nervous system and is also important for inequity aversion. One study using the ultimatum game found the administration of benzodiazepine, which increases the efficacy of GABA at the GABA A receptor, reduces the rejection ratio and activity in the amygdala, dACC and mPFC in response to unfair offers [2]. Like *OXTR*, polymorphisms of the genes coding the subunits of the GABA A receptor were reported to be correlated with altruism [37] and autism [38]. It was also shown that a polymorphism on the promoter region of the enzyme for GABA synthesis modulates ACC activity in humans [39]. On the other hand, many GWAS (Genome Wide Association Studies) have shown that the influence of each single nucleotide polymorphism is small and that most reported genetic associations could be false positives [40–41]. However, some GWAS indicated that there is an association between social traits and genetic variants [42]. In particular, Linnér and colleagues [43] suggested that the genes involved in GABAergic neurotransmission influence personality traits.

In addition, the influence of the GABA A receptor on oxytocin was recently reported in rodents. Blockage of the receptor suppressed the effects of oxytocin on freezing behavior as well as amygdala activity in fear conditioning [26,44]. Sabihi and colleagues [45] showed that the administration of oxytocin to the mPFC was accompanied by increased activation of GABA neurons through the GABA A receptor in the mPFC and altered neuronal activation of the amygdala following the anxiety test [45].

Based on these previous studies, we hypothesized that GABA may also interact with the function of oxytocin in inequity aversion. To address this issue, we conducted a model-based fMRI study of the ultimatum game, a widely-used task in inequity-aversion literature, and quantified the effects of single nucleotide polymorphisms (SNPs) and interactions of oxytocin receptor gene (*OXTR*) and GABA-related genes on human behaviors and brain activities in inequity aversion. We focused on the genes for OXTR, GABAA receptor gene clusters (GABA A receptor subunit clusters on chromosomes 5q34-q35, 4p12, 6q14-16 and 15q11-q13), and enzymes for GABA synthesis. In addition, for our analysis, we conducted the Triple-Dominance measure task, which measures a participant’s egalitarianism in resource allocation [46].

## Materials and methods

### Participants

The ethical committees of the National Institute of Information and Communication Technology (NICT), Japan, Tamagawa University, Japan, and University of Tokyo approved this study, and written informed consent to the behavioral, saliva collection and MRI experiments was obtained from all participants before the experiments were done. The individual in this manuscript has given written informed consent to publish the face image (PLOS consent form).

Two hundred and fourteen Japanese students (111 males, age = 19.5±0.12; 103 females, age = 19.6±0.12) who did not declare any history of neurological or psychiatric disorders participated in the first saliva sample collection for the SNP analysis and the Triple-Dominance measure task to identify their social value orientation (i.e., prosocial, individualistic or competitor). All participants were invited to the fMRI experiments. Adjusting for the availability of the participants and MRI scanning slots, 97 participants (56 males, age = 19.3±0.17; 41 females, age = 19.4±0.22) took part in the fMRI experiments.

### Tasks

#### Triple-Dominance measure task: Day1

**Fig 1:**
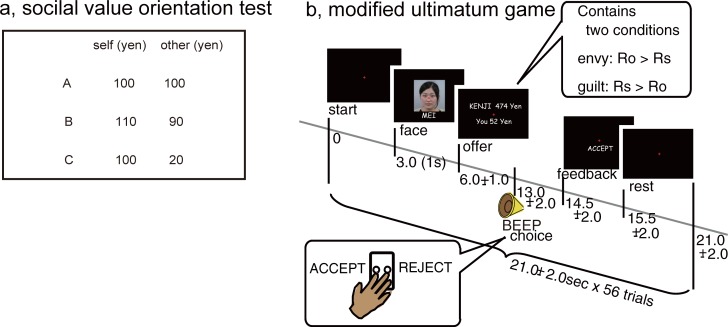
Design of tasks. (A,B) Participants make decisions in (A) the Triple-Dominance Measure task and (B) the modified ultimatum game. Each participant played the role of the responder. The modified ultimatum game contained both advantageous (guilt) and disadvantageous (envy) proposals unlike the standard ultimatum game, which only contains disadvantageous offers.

The Triple-Dominance measure task is a forced three-choice form of money distribution between the self and an unknown other, and has been used to identify a participant’s social value orientation [10–11,46]. Thirteen to forty participants in a room received a sheet of paper on which two numbers were written. One number represented the identity of the participant and the other number the identity of the other participant who was randomly paired with the participant. Participants were presented with 8 Triple-Dominance measure tasks, which asked them to choose the most preferable money distribution for the self and the other from three options within 10 s (Fig 1A). In this particular example, one option (A; prosocial) maximizes the sum of outcomes for the self and other and minimizes the difference of outcomes. It is therefore associated with inequity aversion. A second option (B; individualistic) maximizes the outcome for the self. The third option (C; competitive) maximizes the difference between the outcomes for the self and the other. A participant was assigned a social value orientation (i.e., prosocial, individualist, or competitor) when the participant made more than six consistent choices out of eight. Participants never knew who was paired with them and no feedback was given during the task. Participants received the amount of money based on their choices.

#### Modified ultimatum game: Day2

We used a modified version of the ultimatum game [47] to examine brain responses to inequity (Fig 1B). In comparison with the standard ultimatum game, both disadvantageous (envy; reward for other was larger than reward for self) and advantageous (guilt; reward for self was larger than reward for other) conditions were included. After a short display (1 s) of the name and face of a proposer, the participant was asked to decide whether to accept or reject the offered division of 500 yen (equivalent to 5 US dollars) by a button press within 1 s after a beep. Base offers were one of 7:3, 6:4, 5:5, 4:6, 3:7, 2:8 or 1:9 for the participant (responder) and proposer. Each base offer appeared 8 times in one session in pseudo random order. Therefore, one session comprised 56 trials. Uniform random numbers ranging from -25 yen to 25 yen were added to the base offer in each trial in order to maximize the participant’s involvement in the task. Because each name and face was utilized once, this task was a sequential one-shot game. We instructed the participants that the faces and offers in the ultimatum game were collected from students at a nearby university whose rewards would depend on the participants’ choices. In fact, all faces (neutral facial expressions) were selected from the facial expression database [48] released by the Advanced Telecommunications Research Institute (ATR), Japan. The total time of a session was 1176 s.

Because the ultimatum game is an asymmetric game for a proposer and a responder, rejection behaviors by the responder might partly correspond to costly punishment as well as inequity aversion. Therefore, our SNP analysis considered both the Triple-Dominance measure task and ultimatum game.

### SNP analysis

Saliva sampling was done using the DNA collection kit Oragene·DNA (OG-500) (DNA Genotek Inc.). Using commercially available TaqMan probes and ABI PRISM 7900HT and following the protocol recommended by the manufacturer (Thermo Fisher Scientific, Waltham, MA, USA), we selected and genotyped the SNPs of genes for enzymes involved in GABA synthesis and major subunits of the GABA A receptor: rs3791878 and rs2236418 (GAD1 and GAD2, respectively), and rs3811991, rs2617503, rs1912960, rs2351299, rs279858, rs9362632, rs140682 and rs878960 (GABA A receptor subunit genes clusters on chromosomes 5q34-q35, 4p12, 6q14-16 and 15q11-q13). We did the same for the oxytocin-receptor (OXTR) genes: *rs*237924, *rs*75775, *rs*4686302, *rs*1042778 and *rs*53576 (SNPs upstream of the gene, on the protein coding region, exon and long intron). We divided the genotypes of these SNPs into two groups by combining heterozygotes and minor allele homozygotes, since the frequencies of some minor alleles were not sufficiently high.

### Statistical analysis

We conducted statistical tests of the genetic effects by using the functions ‘ranksum’ and ‘anovan’ in MATLAB R2014a, and the correction for multiple comparisons by using the Benjamini-Hochberg method [49] in R version 3.0.2 (https://www.r-project.org). For the analysis of interactions, a representative SNP on each GABA A receptor cluster was included in ANOVA.

### MRI acquisition

MRI scanning was conducted with a Siemens Trio TIM 3T scanner at Tamagawa University (Japan). The parameters used were: repetition time 2 s, echo time 25 ms, flip angle 90°, field of view 192 mm, and resolution 3 × 3 × 3 mm. High-resolution (T1 [1 × 1 × 1 mm] and T2 [0.6 × 0.4 × 3 mm]) structural images were also acquired for each participant. In addition to the experimental trials, the session contained three initial dummy scans.

### GLM analysis

Imaging data were analyzed using standard procedures in Statistical Parametric Mapping (SPM12 http://www.fil.ion.ucl.ac.uk/spm) on MATLAB R2014a. Before the analysis, we performed motion correction and non-linear transformation into the standard space of the Montreal Neurological Institute (MNI) coordinates using a T2 template. These normalized EPI images were re-sliced into 2 × 2 × 2 mm voxels and then smoothed with a 6 mm FWHM isotropic Gaussian kernel. The data were high-passed filtered (cut-off frequency, 128 s).

First-level analysis: In the main analysis, for each participant, eight functional regressors were included in the general linear model analysis of the fMRI data. The standard event-related regressors were constructed at the time of the proposer’s face presentation, offer presentation, button press (choice) and feedback presentation. For the offer presentation, four reward-related regressors (parametric modulators) were also included: reward for self (Rs), reward for proposer (Ro), envious difference (the reward difference when Rs < Ro), and guilty difference (the reward difference when Ro < Rs). Since the range of the reward variables is continuous and wide (i.e., between 0 and 450), common logarithm (base = 10) was used for these four parametric modulators. In addition to these eight regressors, we included six head movement parameters that were calculated from the realignment.

Second-level analysis: To contrast neural correlates with envy and guilt aversions for polymorphisms, we conducted a second-level group analysis using a multiple-regression for analyzing the effects of polymorphisims on the *OXTR* and GABA-related genes, and using a full factorial design to analyze the interaction between the genotypes of *GAD1 rs*3791878 (GG, GT/ TT) and *OXTR rs*53576 (AA, AG/GG). The neural correlates of envy and guilt aversions were defined as brain activity correlated with the envious reward difference between other and self (Ro-Rs > 0) and with the guilty reward difference between self and other (Rs-Ro > 0), respectively.

### Utility function

Three utility functions were considered to analyze envy and guilt aversions as described below.

$$U=\beta_{self}*R_{s}+\beta_{envy}*(max(R_{o}-R_{s}),0)+\beta_{guilt}*(max(R_{s}-R_{o}),0)$$ Eq. 1

$$U=\beta_{self}*R_{s}+\beta_{inequity}*abs(R_{o}-R_{s})$$ Eq. 2

$$U=\beta_{self}*R_{s}+\beta_{other}*R_{o}+\beta_{inequity}*abs(R_{o}-R_{s})$$ Eq. 3

The weights (β) in the equations were estimated from behaviors during the ultimatum game by the maximum likelifood estimation (MLE) using the internal point method in MATLAB R2014a. Eq 1 comes from Fehr & Schmidt [50]. Envious reward differences and guilty reward differences contribute to judgements separately in this model. Eq 2 and Eq 3 come from the inequity-aversion model, which represents the reward difference (containing both envious and guilty differences) by a single term. The amount of the reward for others is considered in Eq 3, but not in Eq 2. β_self_ was set to 1 in all three equations.

### ROIs for small volume correction

We used the functional ROIs defined by Shen and colleagues when we conducted small volume corrections, which were produced from the resting-state fMRI data of 79 healthy participants and parcellated by group-wise graph theory-based analysis [51]. These ROIs have functional homogeneity within each node and good parcellation reproducibility across multiple groups of healthy volunteers. To select the ROIs for small volume correction, we defined two criteria: 1) previous reports showed the importance of the regions for the inequity aversion or decisions in the ultimatum game (e.g. amygdala and dACC) and 2) the whole brain analysis detected correlation between activity and the inequity aversion or decision (the statistical threshold was *p* < 0.001 uncorrected).

## Results

### Genotype distribution

The genotype distributions of the 97 fMRI-experiment participants are shown in S1 Table. The distributions of all SNPs we examined were not different from the Hardy-Weinberg equilibrium (*p* > 0.01), yielding a result consistent with previous reports [52–53] and datasets, including the HapMap (international HapMap Project) and 1000 Genome project for Asian populations.

### Egalitarianism and SNPs on *OXTR* and GABA-related genes

The effects of oxytocin on prosocial behaviors such as trust and generosity have been previously reported [13,17,24,29]. For egalitarianism, a previous study reported no significant correlation between the donation money and an *OXTR* genetic variation in the dictator game [54]. However, Israel and colleagues [31] reported correlations between SNPs on the long intron region of *OXTR* and social value orientation using the Triple-Dominance measure task. Therefore, we first conducted the Triple-Dominance measure task (Fig 1A) and evaluated the effects of *OXTR* SNPs on egalitarianism. As shown in Table 1, *rs*53576 and *rs*4686302 was significantly associated with the number of prosocial choices (*p* = 0.0027 and 0.0373, respectively, N-way ANOVA). We examined the correlation between social value orientation and *OXTR* SNPs and found that only *rs*53576, an SNP located at the long intron, was significantly correlated with the type of social value orientation (prosocial or individualist; Table 2, *p* = 0.046, Chi-squared test).

**Table 1:** Correlation of the SNPs on *OXTR* and the number of prosocial choices in social value orientation test.

SNP	P	F	test
**rs53576**	0.0027	9.53	N-way ANOVA
**rs4686302**	0.037	4.47	
**rs75775**	0.33	0.94	
**rs237924**	0.57	0.33	
**rs1042778**	0.31	1.06	

**Table 2:** Interactions between Social Value Orientation (SVO) and SNPs on *OXTR*.

SNP	Location	Type	P	
**rs53576**	3^rd^ intron	SNV^a^	0.046	AA/AG.GG
**rs4686302**	3^rd^ exon	SNV(missense)	0.93	CC/CT.TT
**rs75775**	Upstream	SNV	0.45	GG/GT.TT
**rs237924**	Upstream	SNV	0.45	CC/CT.TT
**rs1042778**	Downstream 3’-UTR^b^	SNV	0.55	GG/GT.TT

^a^SNV, single nucleotide variance

^b^UTR, untranslated region.

We also examined relationships between the genotypes of SNPs of GABA-related genes and social value orientation, because a previous study showed that the injection of benzodiazepine, which facilitates the GABA A receptor, decreased the rejection rates of unfair offers in the ultimatum game without changing sensitivity to fairness [2]. However, we found neither a significant effect of SNPs of GABA-related genes (S2 Table) nor an interactive effect between SNPs of GABA-related genes and *rs*53576 of *OXTR* on social value orientation. These results suggested that only *rs*53576 had a main effect on the sensitivity to egalitarianism.

### Behaviors in the ultimatum game

In the context of economic games, inequity aversion can be decomposed into envy (disadvantageous; rewards for others are higher than for self) and guilt (advantageous; rewards for self are higher than for others) aversions [50]. To quantify inequity aversion, the weights for inequity aversion were estimated and compared. We introduced three models (*Eqs1* to *3* in Materials and Methods) and, upon applying the Akaike information criterion (AIC) [55] and the Bayesian information criterion (BIC) [56] (S3 Table), found that the envy-guilt model (*Eq 1*) was most suitable for the present study based on the modified ultimatum game (Fig 1B). Because -β_envy_ and -β_guilt_ are indices that correspond to behavioral decisions based on envy and guilt aversions, we defined the Decision Index (DI) for envy and guilt as -β_envy_ and -β_guilt_, respectively, for the following analyses. The ‘minus’ sign of β means that the feeling is negative (aversive).

### Brain activities in the ultimatum game

**Fig 2:**
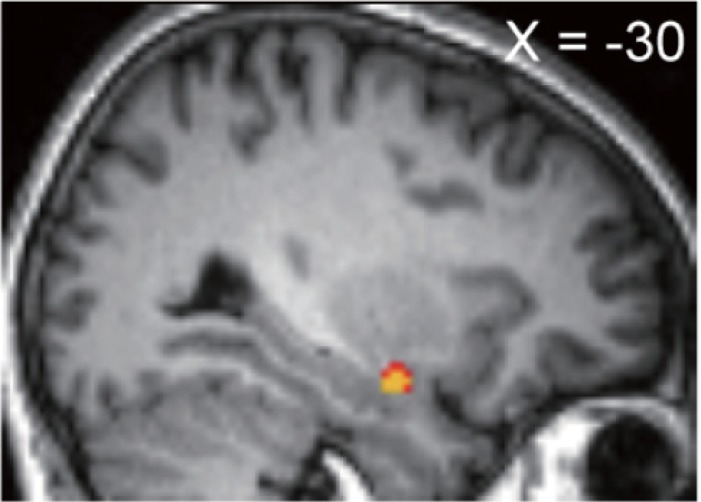
Activation in amygdala correlated with DI_envy_. Amygdala responses to disadvantageous inequity (envy) were correlated with each participant’s DI_envy_ (*P*_*FWE_SVC*_ = 2.9 x 10^−2^ small volume corrected, peak MNI coordinate -30, -6, -18).

To identify the brain activities that correlated with the decisions induced by envy and guilt aversions, we conducted second-level GLM analysis of the brain activations that correlated with envy (Ro > Rs) and guilt (Rs > Rs) using DI_envy_ and DI_guilt_ as second-level regressors (Fig 2). DI_envy_ was found to be correlated with envy-correlated activity in the amygdala (Table 3; *p* = 2.9 x 10^−2^, small volume corrected, MNI coordinates -30, -6, -18), consistent with previous studies [10–11,57]. On the other hand, we did not find any brain activity correlated with DI_guilt_.

**Table 3:** The effects of DI_envy_ on whole brain envy-correlated activity.

Region	peak position^a^			t score	z score	P_unc_^b^	P_FWE_svc_^c^
	x	y	z				
**amygdala**	-30	-6	-18	4.02	3.86	5.8 x 10^−5^	2.9 x 10^−2^

^a^Peak locations are shown as MNI coordinates.

^b^*p* values at the peak are shown.

^c^svc; small volume correction.

### Solitary effects of SNPs on envy aversion

**Fig 3:**
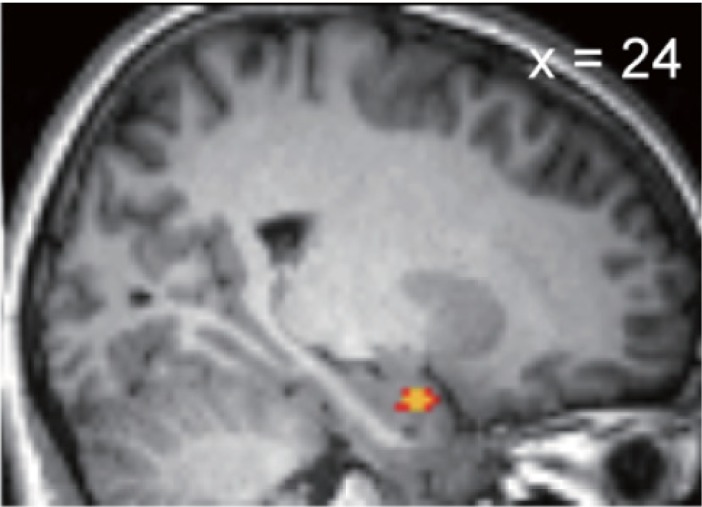
Whole-brain effects of the polymorphisms on *OXTR* (*rs*53576) on envy-correlated activity. Regression analysis showed that amygdala activity induced by inequity was correlated with *rs*53576 ‘A’. (*P*_*FWE_SVC*_ = 1.0 x 10^−2^, peak MNI coordinates 24, -2, -22). The cluster was small-volume corrected using the ROI defined by Shen *et al*. [50], and the threshold of the image was *p* < 0.001 and *p* < 0.005 (uncorrected, yellow and red, respectively) for display purposes.

We looked for the effects of SNPs on DI_envy_. Neither significant solitary (main) effects of SNPs nor interactive effects of SNPs with gender [20,58] were identified (Panels A-B in S4 Table; Wilcoxon rank-sum test). However, the envy-induced amygdala activity was affected by *rs*53576. More specifically, amygdala activity was found to be larger in A carriers of *rs*53576 (Fig 3 and Table 4; *p* = 1.0 x 10^−2^ at the peak position, small volume corrected, MNI coordinates 24, -2, -22). There were no other significant effects of single SNPs on envy-induced brain responses.

**Table 4:** The effects of rs53576 (*OXTR*) on whole brain envy-correlated activity.

Region	peak position^a^			t score	z score	P_unc_^b^	P_FWE_svc_^c^
	x	y	z				
**amygdala**	24	-2	-22	4.36	4.15	1.7 x 10^−5^	1.0 x 10^−2^

^a^Peak locations are shown as MNI coordinates.

^b^*p* values at the peak are shown.

^c^svc, small volume correction.

### Effects of *GAD1*-*OXTR* interaction on envy aversion

**Fig 4:**
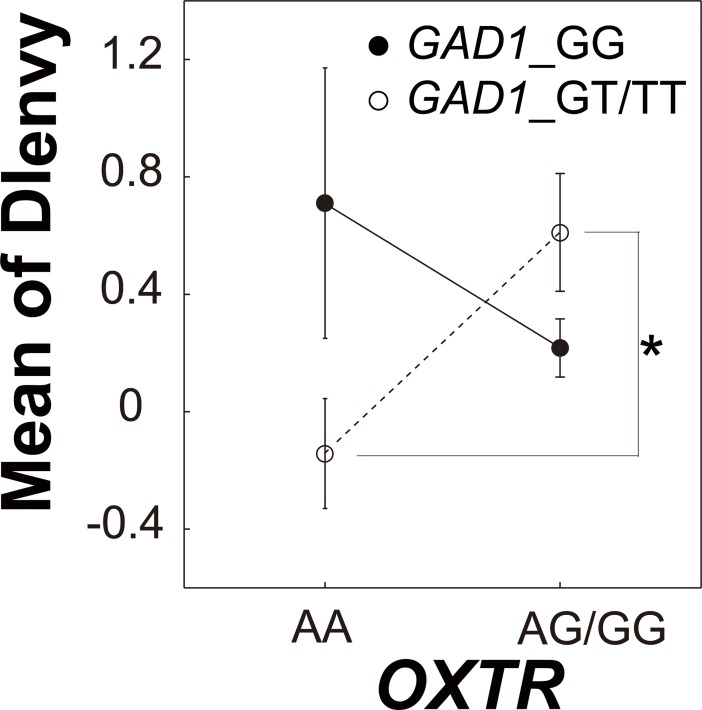
Mean values of the DI_envy_. An interaction between the polymorphisms of *GAD1* (*rs*3791878: GG, filled solid; GT/TT, open dashed) and *OXTR* (*rs*53576) was revealed by ANOVA (*p* = 3.9 x 10^−3^, Table 5). DI_envy_ was larger in *rs*3791878GT/TT-*rs*53576AG/GG carriers than in *rs*3791878GT/TT-*rs*53576AA carriers (asterisk, *p* = 4.2 x 10^−2^, S5 Table). Error bars represent standard errors.

Motivated by reports on oxytocin-GABA interactions in rodents [26,45,59], we investigated the interactive effects between *OXTR* and SNPs of GABA-related genes on DI_envy_. Because *rs*53576 was the only gene correlated with social value orientation and the envy-induced amygdala activity, we chose it as the candidate SNP of *OXTR*. We found significant interactive effects of SNPs (*rs*3791878 (*GAD1*)-*rs*53576 (*OXTR*), *rs*2236418 (*GAD2*)-*rs*140682 (chr 15q), *rs*1912960 (chr 4p)-*rs*53576 (*OXTR*), *rs*1912960 (chr 4p)-gender, and *rs*9362632 (chr 6q)-gender) on DI_envy_ by using the N-way ANOVA (Table 5). We then compared DI_envy_ among the participant groups, which were determined by the SNP subtype in a post-hoc manner (Panels A-E in S5 Table). We found a significant interactive effect between *GAD1* and *OXTR* (Fig 4 and Panel A in S5 Table; *p* = 4.2 x 10^−2^, Wilcoxon ranksum test, correction for multiple comparisons by the number of comparisons in each condition (= 6) was done by the Benjamini and Hochberg method), but not between any other combination (Panels B-E in S5 Table). This result indicates that GABA synthesis and oxytocin presence coordinately modulate the behavioral decisions induced by envy aversion.

**Table 5:** Effects of SNPs or gender on DI_envy_.

Interactive effects between SNPs and gender			
SNP	*p*	F	Test
**rs3791878 (*GAD1*) x rs2236418 (*GAD2*)**	0.87	0.03	N-way ANOVA
**rs3791878 (*GAD1*) x rs3811991 (chr5q)**	0.79	0.07	
**rs3791878 (*GAD1*) x rs1912960 (chr4p)**	0.18	1.81	
**rs3791878 (*GAD1*) x rs9362632 (chr6q)**	0.98	0	
**rs3791878 (*GAD1*) x rs140682 (chr15q)**	0.99	0	
**rs3791878 (*GAD1*) x rs53576 (*OXTR*)**	0.0039	9.05	
**rs3791878 (*GAD1*) x gender**	0.30	1.1	
**rs2236418 (*GAD2*) x rs3811991 (chr5q)**	0.52	0.42	
**rs2236418 (*GAD2*) x rs1912960 (chr4p)**	0.072	3.36	
**rs2236418 (*GAD2*) x rs9362632 (chr6q)**	0.21	1.62	
**rs2236418 (*GAD2*) x rs140682 (chr15q)**	0.045	4.2	
**rs2236418 (*GAD2*) x rs53576 (*OXTR*)**	0.29	1.13	
**rs2236418 (*GAD2*) x gender**	0.43	0.64	
**rs3811991 (chr5q) x rs1912960 (chr4p)**	0.64	0.21	
**rs3811991 (chr5q) x rs9362632 (chr6q)**	0.29	1.16	
**rs3811991 (chr5q) x rs140682 (chr15q)**	0.77	0.08	
**rs3811991 (chr5q) x rs53576 (*OXTR*)**	0.22	1.56	
**rs3811991 (chr5q) x gender**	0.94	0.01	
**rs1912960 (chr4p) x rs9362632 (chr6q)**	0.72	0.13	
**rs1912960 (chr4p) x rs140682 (chr15q)**	0.052	3.95	
**rs1912960 (chr4p) x rs53576 (*OXTR*)**	0.031	4.92	
**rs1912960 (chr4p) x gender**	0.042	4.35	
**rs9362632 (chr6q) x rs140682 (chr15q)**	0.45	0.57	
**rs9362632 (chr6q) x rs53576 (*OXTR*)**	0.21	1.60	
**rs9362632 (chr6q) x gender**	0.028	5.08	
**rs140682 (chr15q) x rs53576 (*OXTR*)**	0.11	2.63	
**rs140682 (chr15q) x gender**	0.13	2.27	
**rs53576 (*OXTR*) x gender**	0.32	1.00	

**Fig 5:**
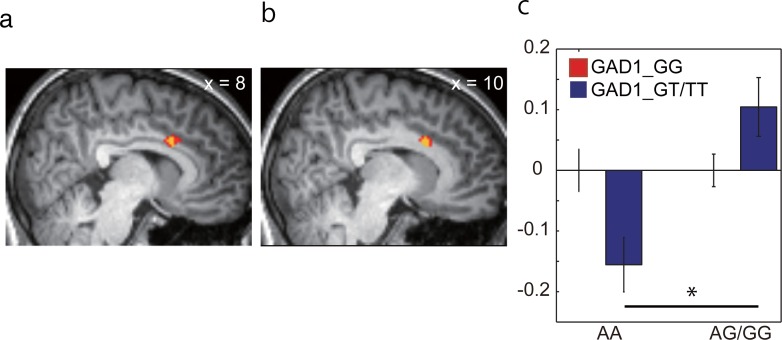
Whole-brain interactive effects between polymorphisms of *GAD1* (*rs*3791878) and *OXTR* (*rs*53576) on envy-correlated activity. (A,B) An interactive effect was found in the dACC (A, *F* = 17.02, *P*_*FWE_SVC*_ = 4.3 x 10^−2^, MNI coordinates 8, 14, 28). More specifically, the response to envy in the dACC was larger in *rs*3791878GT/TT-*rs*53576AG/GG carriers and *rs*3791878GG-*rs*53576AA carriers (B, *P*_*FWE_SVC*_ = 2.8 x 10^−2^). Peak locations in the MNI coordinates are shown in Table 6. Each cluster was small-volume corrected using the ROI defined by Shen *et al*. [50], and the threshold of the image was *p* < 0.001 and *p* < 0.005 (uncorrected, yellow and red respectively) for display purposes. The envy-correlated activity at the peak location in B (MNI coordinates 10, 14, 28) is displayed separately for the different groups shown in Fig 4 (*rs*3791878GT/TT-*rs*53576AG/GG > *rs*3791878GT/TT-*rs*53576AA). (C) Mean envy-induced activities in the *rs*3791878GG and *rs*3791878 GT/TT groups are shown in blue and red, respectively.

We next wished to identify envy-correlated brain activity that paralleled the interactive effect between *GAD1* (GG, GT/TT) and *OXTR* (AA, AG/GG) by conducting a full factorial design analysis (see Materials and Methods). We identified a significant interactive effect in the dACC (Fig 5A and Table 6; *F* = 17.02, *p* = 4.3 x 10^−2^ at the peak position, small volume corrected, MNI coordinates 8, 14, 28). This brain region has been consistently highlighted in inequity aversion [2,11,60].

**Table 6:** Interactive effects between *GAD1* and *OXTR* on whole brain envy-correlated activity.

**All groups**							
**Region**	**peak position^a^**			**F**	**z** **score**	**P_unc_^b^**	**P_FWE_svc_^c^**
	**x**	**y**	**z**				
**dACC**	8	14	30	17.02	3.77	8.0 x 10^−5^	4.3 x 10^−2^
**rs3791878GT/TT-rs53576AG/GG > rs3791878GT/TT-rs53576AA**							
**Region**	**peak position**^**a**^			**t** **score**	**z** **score**	**P**_**unc**_^**b**^	**P**_**FWE_svc**_^**c**^
	**x**	**y**	**z**				
**dACC**	10	14	28	4.04	3.87	5.4 x 10^−5^	2.8 x 10^−2^

^a^Peak locations are shown as MNI coordinates.

^b^*p* values at the peak are shown.

^c^svc, small volume correction.

We further compared the envy-correlated dACC activity between two groups whose DI_envy_ were different (i.e., *rs*3791878GT/TT-*rs*53576AG/GG > *rs*3791878GT/TT-*rs*53576AA). Whole brain analysis revealed that dACC activity was higher in the higher DI_envy_ group (Fig 5B and 5c and Table 6; *p* = 2.8 x 10^−2^ at the peak position, small volume corrected, MNI coordinates 10, 14, 28). These results strongly indicated that the genetic interaction between *GAD1* and *OXTR* had an influence on the envy-induced activation of the dACC.

## Discussion

In this study, we reported the interactive effect between GABA- and oxytocin-related genes on human envy aversion. We found that each participant’s DI_envy_ calculated from accept/reject behavior during the ultimatum game was correlated with the interaction effect between *GAD1* and *OXTR* (Fig 4) and that this interactive effect was correlated with the envy-induced activity of the dACC (Fig 5), which has been implicated to play a crucial role in social information processing [61].

The response to unfair offers consists of at least two process: the manipulation of aversive feelings and the decision-making based on the aversive feelings. In our task, unfair proposals induced aversive feelings. The aversive feelings were larger in people with prosocial traits. We confirmed that the correlation between social value orientation and the polymorphism on *OXTR* (Table 1 indicated that *rs*53576 ‘A’ correlated with the prosocial trait). The aversive feelings induced by inequity were previously reported to correlate with the amygdala response to inequity [10,11], and we found the correlation between the type of *OXTR* and the amygdala activity correlated with envy aversion (inequity) (Fig 3). These findings suggested contributions by oxytocin and the amygdala in the first process.

On the other hand, in the second process, decisions (accept/reject) were made by taking aversive feelings into consideration. In our procedure, decision indices calculated from the rejection behaviors in the envy condition correlated with the *OXTR*-*GAD1* interaction (Fig 4). This observation suggested that envy aversion depends on interactive effects between the sensitivity to inequity (which was related to the type of *OXTR*) and the function of GABA (which was related to the type of *GAD1*). We also found that this interactive effect was correlated with envy-related brain activity in the dACC (Fig 5). In this task, participants had to compensate aversive feelings to accept envious proposals, and this discrepancy between inequity aversion and the accepted decision was larger in prosocials who disliked inequity (*rs*53576 ‘A’). Researchers have repeatedly reported the contribution of the dACC to resolving conflicts [62,63]. Especially in the context of the ultimatum game, it was suggested that activity in the dACC decreases when participants forgive unfair partners [64]. Our results may suggest that the dACC activity controlled by *GAD1* has an effect on resolving conflicts between inequity aversion and the accepted decision. This hypothesis is consistent with a report that shows correlation between the polymorphism of the *GAD1* gene and the change in GABA concentration in human dACC [65].

It was reported that the administration of oxytocin in humans changes the anxiety trait [66] and that the anxiety trait is related to the microstructural property of the amygdala-ACC pathway [67]. The GABA concentration in the dACC was reported to correlate with amygdala activation during the processing of emotional stimuli [68]. Our study may extend these findings and suggest the possibility that the amygdala activity in social tasks is generally linked with GABA levels in the dACC.

Animal studies have indicated that oxytocin neurons project to both the amygdala and ACC [26]. OXTR is also expressed in both the amygdala and ACC in humans [69]. Although it is difficult for the present study to clarify whether oxytocin works on the ACC directly or indirectly through the amygdala, we found that oxytocin contributes to the aversive feelings to inequity that are mainly expressed in the amygdala, while the interaction between oxytocin and GABA synthesis affects the decision-making that is based on inequity aversion, which is principally represented in the dACC. This observation is comparable with a previous report that showed rejection behavior was not influenced by oxytocin administration [24].

Several studies have reported that the GABA A receptor is essential for oxytocin function in fear and anxiety conditions [26,45]. In the present study, no SNPs of GABA A receptor-related genes had an interactive effect with the SNP of *OXTR*, but the SNP of *GAD1* did. One potential explanation for this observation is that the GABA A receptor consists of five subunits, each encoded by a distinct gene. SNPs of the individual subunit genes might have only a small effect, as Benjamin and colleagues [40] stated, while *GAD1* encodes the enzyme that synthesizes GABA and has a direct effect on the amount of GABA that could control neural activities.

With respect to the SNPs of *OXTR*, several contradictory results have been reported between Asian and Caucasian populations regarding the long intron region [32–34]. We reported here that the ‘A’ allele of *r*s53576 was correlated with the prosocial trait in Japanese, but the ‘A’ allele was correlated with the antisocial trait in Caucasians [70]. Therefore, the SNP itself may not be the real cause of the phenotype variation. Differences in social culture or physical environment might account for the opposite effects of the same allele type between different populations. Since all participants in our experiments were Japanese university students, we could not assess regional or age differences. Regarding gender differences, we did not find a gender difference in the present study (neither social value orientation nor decision indices in the envy condition) despite contradictory evidence regarding the effects of intranasal oxytocin injection and *OXTR* polymorphisms in social contexts [20,71–74]. However, we did find a significant correlation between the guilt decision index and the type of *OXTR* in females (*p* = 0.025, Wilcoxon ranksum test, correction for multiple comparisons by the number of comparisons in each condition was done by the Benjamini and Hochberg method). This finding may indicate not only that the effect of oxytocin on inequity aversion is different for envy and guilt conditions, but also that the effect of oxytocin on guilty feelings is different between males and females. Further studies are necessary to validate this hypothesis.

## Supporting information

S1 Table Genotype distributions. Distributions of the SNPs are shown.
(DOCX)

S2 Table Interactions between SVO and SNPs on GABA-related genes. There was no interactive effect between SNPs of GABA-related genes.
(DOCX)

S3 Table Model selection by the Akaike or Bayesian information criterion (x 10^4^). The envy-guilt model (*Eq 1*) was most suitable for the present study based on the modified ultimatum game.
(DOCX)

S4 Table Effects of SNPs or gender on DI_envy_. No significant solitary (main) effects of SNPs nor interactive effects of SNPs with gender were identified.
(DOCX)

S5 Table Interactive effects of subtypes and gender on DI_envy_. We found a significant interactive effect between *GAD1* and *OXTR*.
(DOCX)
