# Supplementary material for: Interactive effects of OXTR and GAD1 on envy-associated behaviors and neural responses
Source: PLoS One. 2019 Jan 11;14(1):e0210493. doi: 10.1371/journal.pone.0210493 (PMC6329522; doi:10.1371/journal.pone.0210493)
Supplement: S1 Table — Distributions of the SNPs are shown. (DOCX) [file pone.0210493.s001.docx]

**S1 Table. Genotype distribution.**

| **SNP** | **Participants** | | | **HapMap** |
| --- | --- | --- | --- | --- |
|  | **Number** | **HWE^a^** | |  |
|  |  | **χ^2^** | ***P*** |  |
| **rs3791878** | GG/GT/TT | 1.69 | 0.19 | ss70455802 |
| **(*GAD1*)** | 65/31/1 |  |  | 62.3/34.1/0.035 |
| **rs2236418** | AA/AG/GG | 0.078 | 0.78 | ss76888352 |
| **(*GAD2*)** | 32/48/16 |  |  | 29.1/48.8/22.1 |
| **rs3811991** | AA/AC/CC | 0.53 | 0.47 | ss61715243 |
| **(chr5q34-q35)** | 46/38/11 |  |  | 44.2/45.3/10.5 |
| **rs2617503** | CC/CT/TT | 0.14 | 0.71 | ss44634250 |
| **(chr5q34-q35)** | 46/38/11 |  |  | 20.5/54.5/25.0 |
| **rs1912960** | GG/CG/CC | 0.30 | 0.59 | - |
| **(chr4p12)** | 34/49/14 |  |  |  |
| **rs2351299** | GG/GT/TT | 0.73 | 0.39 | ss44520803 |
| **(chr4p12)** | 39/48/10 |  |  | 36.5/57.6/0.059 |
| **rs279858** | CC/CT/TT | 0.75 | 0.39 | ss5287992 |
| **(chr4p12)** | 23/44/30 |  |  | 17.4/38.4/44.2 |
| **rs9362632** | CC/CG/GG | 0.087 | 0.77 | ss22499528 |
| **(chr6q14-16)** | 54/36/7 |  |  | 48.9/46.7/4.4 |
| **rs140682** | CC/CT/TT | 0.93 | 0.34 | - |
| **(chr15q11-q13)** | 45/39/13 |  |  |  |
| **rs878960** | CC/CT/TT | 0.24 | 0.62 | ss1304493 |
| **(chr15q11-q13)** | 30/50/17 |  |  | 37.2/41.9/20.9 |
| **rs53576** | AA/AG/GG | 0.0023 | 0.96 | - |
| **(*OXTR*)** | 36/46/15 |  |  |  |
| **rs4686302** | CC/CT/TT | 0.00036 | 0.77 | - |
| **(*OXTR*)** | 58/34/5 |  |  |  |
| **rs75775** | GG/GT/TT | 0.037 | 0.55 | - |
| **(*OXTR*)** | 28/48/19 |  |  |  |
| **rs237924** | CC/CT/TT | 0.38 | 0.55 | - |
| **(*OXTR*)** | 28/51/18 |  |  |  |
| **rs1042778** | GG/GT/TT | 0.021 | 0.89 | - |
| **(*OXTR*)** | 77/19/1 |  |  |  |

^a^HWE, Hardy-Weinberg equilibrium.
