# Supplementary material for: Interactive effects of OXTR and GAD1 on envy-associated behaviors and neural responses
Source: PLoS One. 2019 Jan 11;14(1):e0210493. doi: 10.1371/journal.pone.0210493 (PMC6329522; doi:10.1371/journal.pone.0210493)
Supplement: S2 Table — There was no interactive effect between SNPs of GABA-related genes. (DOCX) [file pone.0210493.s002.docx]

**S2 Table. Interactions between SVO and SNPs on GABA related genes.**

| **SNP** | **Location** | **Type** | **P** | **genotype** |
| --- | --- | --- | --- | --- |
| **rs3791878** | Chr2q (*GAD1*) | SNV^a^ | 0.52 | GG/GT.TT |
| **rs2236418** | Chr10p (*GAD2*) | SNV | 0.77 | AA/AG.GG |
| **rs3811991** | Chr5q (*GABRA6*) | SNV | 0.92 | AA/AC.CC |
| **rs2617503** | Chr5q (*GABRB2*) | SNV | 0.95 | CC/CT.TT |
| **rs1912960** | Chr4p (*GABRA4*) | SNV | 0.16 | CC/CG.GG |
| **rs2351299** | Chr4p (*GABRB1*) | SNV | 0.50 | GG/GT.TT |
| **rs279858** | Chr4p (*GABRA2*) | SNV | 0.83 | CC/CT.TT |
| **rs9362632** | Chr6q (*GABRR2*) | SNV | 0.69 | CC/CG.GG |
| **rs140682** | Chr15q (*GABRA5*) | SNV | 0.86 | CC/CT.TT |
| **rs878960** | Chr15q (*GABRB3*) | SNV | 1 | CC/CT.TT |

^a^SNV, single nucleotide variance; UTR, untranslated region.
