# Supplementary material for: Interactive effects of OXTR and GAD1 on envy-associated behaviors and neural responses
Source: PLoS One. 2019 Jan 11;14(1):e0210493. doi: 10.1371/journal.pone.0210493 (PMC6329522; doi:10.1371/journal.pone.0210493)
Supplement: S3 Table — The envy-guilt model (Eq 1) was most suitable for the present study based on the modified ultimatum game. (DOCX) [file pone.0210493.s003.docx]

**S3 Table. Model selection by the Akaike or Bayesian information criterion (x 10^4^).**

| **Model** | **AIC** | **BIC** |
| --- | --- | --- |
| **envy-guilt model (*Eq.1*)** | 3.99 | 4.03 |
| **absolute-inequity model (*Eq.2*)** | 7.36 | 7.39 |
| **reward-quantity model (*Eq.3*)** | 7.98 | 8.02 |
