# Supplementary material for: Interactive effects of OXTR and GAD1 on envy-associated behaviors and neural responses
Source: PLoS One. 2019 Jan 11;14(1):e0210493. doi: 10.1371/journal.pone.0210493 (PMC6329522; doi:10.1371/journal.pone.0210493)
Supplement: S4 Table — No significant solitary (main) effects of SNPs nor interactive effects of SNPs with gender were identified. (DOCX) [file pone.0210493.s004.docx]

**S4 Table. Effects of SNPs or gender on DI_envy_.**

| **(A) Solitary effects of SNPs and gender** | | | |
| --- | --- | --- | --- |
| **SNP** | ***p*** | **F** | **Test** |
| **rs3791878 (*GAD1*)** | 0.62 | 0.24 | N-way ANOVA |
| **GG vs. GT/TT** |  |  |  |
| **rs2236418 (*GAD2*)** | 0.23 | 1.50 |  |
| **AA vs. AG/GG** |  |  |  |
| **rs3811991 (chr5q)** | 0.42 | 0.66 |  |
| **AA vs. AC/CC** |  |  |  |
| **rs2617503 (chr5q** | 0.46 | 0.56 |  |
| **CC vs. CT/TT** |  |  |  |
| **rs1912960 (chr4p)** | 0.15 | 2.14 |  |
| **CC vs. CG/GG** |  |  |  |
| **rs2351299 (chr4p)** | 0.80 | 0.07 |  |
| **GG vs. GT/TT** |  |  |  |
| **rs279858 (chr4p** | 0.35 | 0.90 |  |
| **CC/CT vs. TT** |  |  |  |
| **rs9362632 (chr 6q)** | 0.54 | 0.37 |  |
| **CC vs. CG/GG** |  |  |  |
| **rs140682 (chr15q)** | 0.18 | 1.81 |  |
| **CC vs. CT/TT** |  |  |  |
| **rs878960 (chr15q)** | 0.84 | 0.04 |  |
| **CC vs. CT/TT** |  |  |  |
| **rs53576 (*OXTR*)** | 0.83 | 0.05 |  |
| **AA vs. AG/GG** |  |  |  |
| **rs1042778(*OXTR*)** | 0.97 | 0 |  |
| **GG vs. GT/TT** |  |  |  |
| **rs237924(*OXTR*)** | 0.15 | 2.15 |  |
| **CC vs. CT/TT** |  |  |  |
| **rs75775(*OXTR*)** | 0.64 | 0.22 |  |
| **GG vs. GT/TT** |  |  |  |
| **rs4686302(*OXTR*)** | 0.98 | 0 |  |
| **CC vs. CT/TT** |  |  |  |
| **Gender** | 0.22 | 1.51 |  |
| **male vs. female** |  |  |  |

| **(B) Solitary effects of SNPs and gender** | | | |
| --- | --- | --- | --- |
| **SNP** | ***p*** | **Z** | **Test** |
| **rs3791878 (*GAD1*)** | 0.87 | 0.16 | Wilcoxon ranksum test |
| **GG vs. GT/TT** |  |  |  |
| **rs2236418 (*GAD2*)** | 0.70 | -0.38 |  |
| **AA vs. AG/GG** |  |  |  |
| **rs3811991 (chr5q)** | 0.60 | -0.52 |  |
| **AA vs. AC/CC** |  |  |  |
| **rs2617503 (chr5q** | 0.95 | -0.065 |  |
| **CC vs. CT/TT** |  |  |  |
| **rs1912960 (chr4p)** | 0.53 | -0.62 |  |
| **CC vs. CG/GG** |  |  |  |
| **rs2351299 (chr4p)** | 0.96 | -0.044 |  |
| **GG vs. GT/TT** |  |  |  |
| **rs279858 (chr4p** | 0.63 | -0.49 |  |
| **CC/CT vs. TT** |  |  |  |
| **rs9362632 (chr 6q)** | 0.58 | -0.55 |  |
| **CC vs. CG/GG** |  |  |  |
| **rs140682 (chr15q)** | 0.40 | 0.84 |  |
| **CC vs. CT/TT** |  |  |  |
| **rs878960 (chr15q)** | 0.64 | -0.47 |  |
| **CC vs. CT/TT** |  |  |  |
| **rs53576 (*OXTR*)** | 0.15 | 1.45 |  |
| **AA vs. AG/GG** |  |  |  |
| **rs1042778(*OXTR*)** | 0.94 | 0.076 |  |
| **GG vs. GT/TT** |  |  |  |
| **rs237924(*OXTR*)** | 0.63 | -0.48 |  |
| **CC vs. CT/TT** |  |  |  |
| **rs75775(*OXTR*)** | 0.66 | 0.45 |  |
| **GG vs. GT/TT** |  |  |  |
| **rs4686302(*OXTR*)** | 0.22 | 1.22 |  |
| **CC vs. CT/TT** |  |  |  |
| **Gender** | 0.21 | -1.25 |  |
| **male vs. female** |  |  |  |
