# Supplementary material for: Interactive effects of OXTR and GAD1 on envy-associated behaviors and neural responses
Source: PLoS One. 2019 Jan 11;14(1):e0210493. doi: 10.1371/journal.pone.0210493 (PMC6329522; doi:10.1371/journal.pone.0210493)
Supplement: S5 Table — We found a significant interactive effect between GAD1 and OXTR. (DOCX) [file pone.0210493.s005.docx]

**S5 Table. Interactive effects of subtypes and gender on DI_envy_.**

**(A) rs3791878 and rs53576**

| **Groups^a^** | **Wilcoxon ranksum test** | **Benjamini-Hochberg correction** |
| --- | --- | --- |
| **I vs. II** | 0.050 | 0.075 |
| **I vs. III** | 0.40 | 0.41 |
| **I vs. IV** | 0.081 | 0.097 |
| **II vs. III** | 0.039 | 0.075 |
| **II vs. IV** | 0.0070 | 0.042 |
| **III vs. IV** | 0.034 | 0.075 |

^a^I, rs3791878GG-rs53576AA; II, rs3791878GT/TT-rs53576AA; III, rs3791878GG-rs53576AG/GG; IV, rs3791878GT/TT-rs53576AG/GG.

**(B) rs2236418 and rs140682**

| **Groups^a^** | **Wilcoxon ranksum test** | **Benjamini-Hochberg correction** |
| --- | --- | --- |
| **I vs. II** | 0.34 | 0.50 |
| **I vs. III** | 0.26 | 0.50 |
| **I vs. IV** | 0.41 | 0.50 |
| **II vs. III** | 0.22 | 0.50 |
| **II vs. IV** | 0.50 | 0.50 |
| **III vs. IV** | 0.26 | 0.50 |

^a^I, rs2236418AA-rs140682CC; II, rs2236418AA-rs140682CT/TT; III, rs2236418AG/GG-rs140682CC; IV, rs2236418 AG/GG-rs140682CT/TT.

**(C) rs1912960 and rs53576**

| **Groups^a^** | **Wilcoxon ranksum test** | **Benjamini-Hochberg correction** |
| --- | --- | --- |
| **I vs. II** | 0.35 | 0.35 |
| **I vs. III** | 0.24 | 0.35 |
| **I vs. IV** | 0.29 | 0.35 |
| **II vs. III** | 0.072 | 0.34 |
| **II vs. IV** | 0.11 | 0.34 |
| **III vs. IV** | 0.30 | 0.35 |

^a^I, rs1912960GG-rs53576AA; II, rs1912960GC/CC-rs53576AA; III, rs1912960GG-rs53576AG/GG; IV, rs1912960GC/CC-rs53576AG/GG

**(D) rs1912960 and gender**

| **Groups^a^** | **Wilcoxon ranksum test** | **Benjamini-Hochberg correction** |
| --- | --- | --- |
| **I vs. II** | 0.20 | 0.34 |
| **I vs. III** | 0.30 | 0.34 |
| **I vs. IV** | 0.10 | 0.34 |
| **II vs. III** | 0.32 | 0.34 |
| **II vs. IV** | 0.34 | 0.34 |
| **III vs. IV** | 0.19 | 0.34 |

^a^I, rs1912960GG-female; II, rs1912960GG-male; III, rs1912960GC/CC-female; IV, rs1912960GC/CC-male

**(E) rs9362632 and gender**

| **Groups^a^** | **Wilcoxon ranksum test** | **Benjamini-Hochberg correction** |
| --- | --- | --- |
| **I vs. II** | 0.18 | 0.43 |
| **I vs. III** | 0.45 | 0.45 |
| **I vs. IV** | 0.065 | 0.39 |
| **II vs. III** | 0.36 | 0.43 |
| **II vs. IV** | 0.31 | 0.43 |
| **III vs. IV** | 0.24 | 0.43 |

^a^I; rs9362632CC-female, II; rs9362632CC-male, III; rs9362632CG/GG-female, IV; rs9362632CG/GG-male
